# CBAP modulates Akt-dependent TSC2 phosphorylation to promote Rheb-mTORC1 signaling and growth of T-cell acute lymphoblastic leukemia

**DOI:** 10.1038/s41388-018-0507-6

**Published:** 2018-09-28

**Authors:** Yun-Jung Chiang, Wei-Ting Liao, Kun-Chin Ho, Shih-Hao Wang, Yu-Guang Chen, Ching-Liang Ho, Shiu-Feng Huang, Lee-Yung Shih, Hsin-Fang Yang-Yen, Jeffrey Jong-Young Yen

**Affiliations:** 1 Institute of Biomedical Sciences, Academia Sinica, Taipei, 11529 Taiwan; 2 Division of Hematology/Oncology, Department of Internal Medicine, Tri-Service General Hospital, National Defense Medical Center, Taipei, 11490 Taiwan; 3 Institute of Molecular and Genomic Medicine, National Health Research Institutes, Miaoli County, 35053 Taiwan; 4 Division of Hematology-Oncology, Department of Internal Medicine, Chang Gung Memorial Hospital at Linkou, College of Medicine, Chang Gung University, Taoyuan, 33305 Taiwan; 5 Institute of Molecular Biology, Academia Sinica, Taipei, 11529 Taiwan

**Keywords:** Oncogenes, Acute lymphocytic leukaemia

## Abstract

High-frequency relapse remains a clinical hurdle for complete remission of T-cell acute lymphoblastic leukemia (T-ALL) patients, with heterogeneous dysregulated signaling profiles—including of Raf-MEK-ERK and Akt-mTORC1-S6K signaling pathways—recently being implicated in disease outcomes. Here we report that GM-CSF/IL-3/IL-5 receptor common β-chain-associated protein (CBAP) is highly expressed in human T-ALL cell lines and many primary tumor tissues and is required to bolster leukemia cell proliferation in tissue culture and for in vivo leukemogenesis in a xenograft mouse model. Downregulation of CBAP markedly restrains expansion of leukemia cells and alleviates disease aggravation of leukemic mice. Transcriptomic profiling and molecular biological analyses suggest that CBAP acts upstream of Ras and Rac1, and functions as a modulator of both Raf-MEK–ERK and Akt-mTORC1 signaling pathways to control leukemia cell growth. Specifically, CBAP facilitated Akt-dependent TSC2 phosphorylation in cell-based assays and in vitro analysis, decreased lysosomal localization of TSC2, and elevated Rheb-GTP loading and subsequent activation of mTORC1 signaling. Taken together, our findings reveal a novel oncogenic contribution of CBAP in T-ALL leukemic cells, in addition to its original pro-apoptotic function in cytokine-dependent cell lines and primary hematopoietic cells, by demonstrating its functional role in the regulation of Akt-TSC2-mTORC1 signaling for leukemia cell proliferation. Thus, CBAP represents a novel therapeutic target for many types of cancers and metabolic diseases linked to PI3K-Akt-mTORC1 signaling.

## Introduction

T-cell acute lymphoblastic leukemia (T-ALL) is a high-risk leukemia subtype that accounts for 10–15% of pediatric and 25% of adult ALL cases [1]. Although the remission rate has significantly improved over the past decade, T-ALL remains a therapeutic challenge due to the high frequency of induction failure [2] and early relapse, which is mostly resistant to further treatment [3]. Molecular abnormalities that have frequently been reported in T-ALL include activation mutations of Notch1 and JAK1 and inactivation mutations of PTEN and FBXW7 [4]. The PI3K-Akt-mTOR signaling axis is an important contributory pathway for T-cell leukemia [5, 6]. It is frequently upregulated in patients with T-ALL and its activation is correlated with poor prognosis, therapeutic resistance, and disease relapse [5, 7–9]. Deletion of *mTOR complex-1* (*mTORC1*) in a T-ALL mouse model resulted in profound cell-cycle arrest and efficient eradication of T-ALL cells [10, 11], suggesting that mTORC1 can integrate signals from the PI3K-Akt and MEK–ERK signaling pathways to activate downstream components responsible for tumor cell growth and metabolism [12].

Tuberous sclerosis is a hereditary syndrome characterized by hamartoma formation in various tissues, which is caused by mutations in either *TUBEROUS SCLEROSIS COMPLEX 1* (*TSC1*) or *TSC2* genes [13]. The tuberous sclerosis complex (TSC) is typically composed of TSC1, TSC2, and Tre2-Bub2-Cdc16 domain family member 7 (TBC1D7) subunits. It can be regulated through the PI3K-Akt, Ras-ERK-RSK1, LKB1-AMPK, IKKβ, GSK3β, and HIFα-REDD1 signaling pathways, all of which can be activated by several stimuli such as growth factors, inflammation, energy stress, hypoxia, and the Wnt pathway [14, 15]. Thus far, the TSC is the only known direct inhibitor for activity of the small GTPase Ras homolog enriched in brain (Rheb), which is a critical activator for mTORC1 signaling, i.e., the major promoter of cellular growth and metabolism [14, 16–19]. Therefore, the TSC represents a key controller of the Rheb-mTORC1 signaling network, which is commonly activated via upstream signaling dysregulation due to oncogenic mutation of genes or post-translational protein modifications in tumors. Suppression of Rheb-mTORC1 activation is dependent on translocation of the TSC to the lysosomal surface [20, 21].

CBAP, also known as TMEM102 (Gene ID:284114), was first identified as an interacting protein of the GM-CSF/IL-3/IL-5 receptor common β-chain and participates in cytokine deprivation-induced apoptosis [22]. Bioinformatics analyses have revealed that CBAP is a member of the Mab21 subfamily that lies within the nucleotide transferase protein fold superfamily [23]. Our previous studies have demonstrated that CBAP participates in chemokine-enhanced T-cell migration and adhesion [24] and in T-cell receptor engagement-induced phosphorylation of ZAP-70 and PLCγ1 [25]. Since CBAP proteins are highly expressed in many established tumor cell lines, including T-cell leukemia, we examined whether CBAP is also involved in leukemia proliferation and tumorigenesis. By manipulating the expression of the gene encoding CBAP with knockdown/knockout strategies in T-ALL cells, we demonstrate that CBAP participates in tumor cell growth and leukemogenesis in mice. Importantly, we further reveal the underlying mechanism by which CBAP facilitates Akt-mediated suppression of TSC2, which is accompanied by an increase of Rheb-GTP loading and activation of the mTORC1-signaling pathway to promote leukemogenesis.

## Results

### CBAP enhances the growth of leukemia cells

**Fig. 1:**
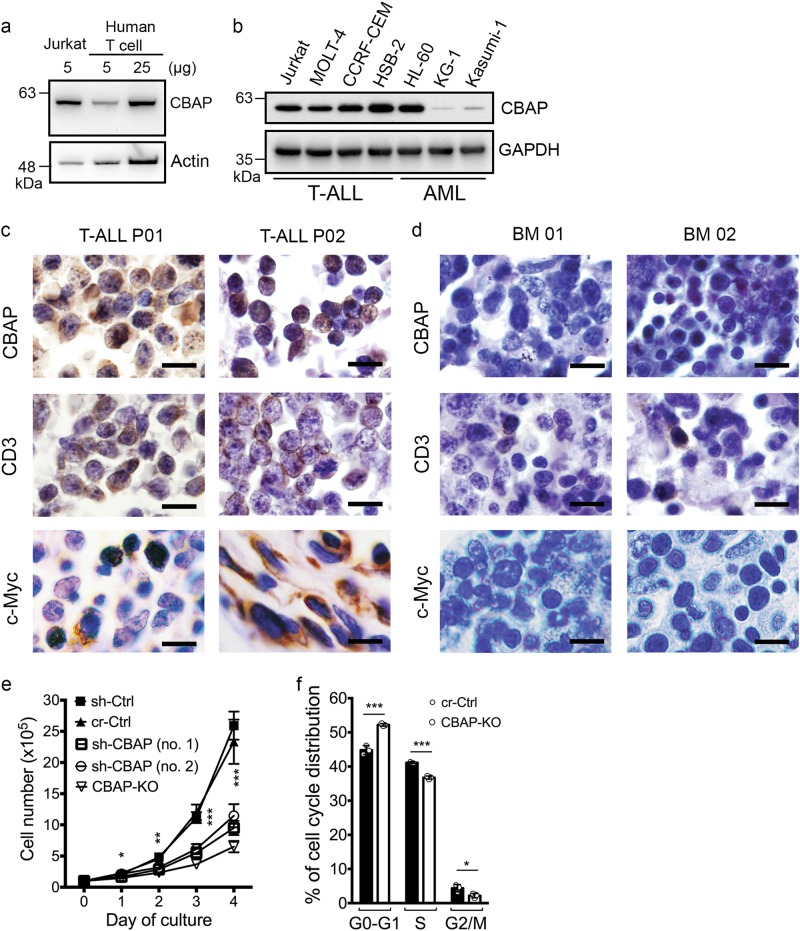
CBAP is highly expressed in T-ALL cell lines and patient cells, and it is important for the regulation of cell proliferation. **a**, **b** Immunoblots of CBAP in human peripheral CD3^+^ T lymphocytes and Jurkat cells (**a**), and in a panel of T-ALL and acute myeloid leukemia (AML) cell lines (**b). c**, **d** IHC staining with CBAP, CD3 and c-Myc antibodies was performed on bone marrow biopsy specimens from two T-ALL patients (T-ALL P01 and P02) (**c**) or two anemia patients (BM 01 and 02) (**d**). Scale bars, 10 μm. **e** Growth of leukemic Jurkat cells stably transduced with lentiviruses expressing control shRNA (sh-Ctrl) or CBAP shRNAs (sh-CBAP no. 1 or no. 2), or clones with (CBAP-KO) or without (cr-Ctrl) CBAP knockout using a CRISPR/Cas9-CBAP-targeting vector. Data are expressed as means ± SD (*n* = 4). **f** Cell-cycle distribution of leukemic Jurkat cells without knockout (cr-Ctrl) or with knockout of CBAP (CBAP-KO), measured by BrdU/7-AAD staining. Data are expressed as means ± SD (*n* = 3). *P < 0.05; **P < 0.01; ***P < 0.001, according to two-tailed unpaired Student *t* tests

We first observed that CBAP protein expression was higher in a Jurkat T-ALL cell line than in purified human peripheral T lymphocytes (CD3^+^ T cells) (Fig. 1a), but these latter conversely expressed a higher level of *CBAP* mRNA than Jurkat T cells (Supplementary Fig. 1a). Interestingly, CBAP protein levels were elevated in all four T-ALL cell lines analyzed, but only in one of the acute myeloid leukemia cell lines we examined (HL60) (Fig. 1b). To confirm this overexpression of CBAP in leukemic cells, we further verified CBAP protein expression in bone marrow (BM) biopsy sections of T-ALL patients (Table 1) by immunohistochemical (IHC) staining. IHC staining for CD3 was positive and diffuse, confirming that most of the tumor cells in the BM sections are T cells (Fig. 1c, middle row), and only a few were positive in the BM sections from control patients (Fig. 1d, middle row). CBAP protein was diffusely overexpressed in T-ALL tumor cells (Fig. 1c, upper row) when compared with the control (anemia patients), with these latter showing no obvious CBAP expression in normal BM biopsy sections (Fig. 1d, upper row). We also assessed C-Myc protein expression as a downstream marker of mTORC1 activation and found strong nuclear C-Myc staining in two T-ALL patients but not in the control patients (Fig. 1c, bottom row). Therefore, we hypothesized that higher CBAP protein expression may confer a beneficial effect on T-ALL cells. To investigate this possibility, we generated a CBAP knockout (KO) Jurkat cell line using the CRISPR/Cas9 technique, and confirmed the absence of CBAP expression by immunoblotting (Supplementary Fig. 1b, left panel). These CBAP-KO Jurkat cells exhibited reduced chemokine-induced migratory activity (Supplementary Fig. 1c), which is consistent with our previous observation [24]. Importantly, CBAP downregulation by either one of two different shRNA knockdown plasmids (sh-CBAP no. 1 and no. 2), or by CRISPR/Cas9 plasmid–mediated KO, caused a significant and consistent reduction in the proliferation rate of Jurkat cells (Fig. 1e), as well as in CCRF-CEM T-ALL cells (Supplementary Figs. 1b, right panel, and 1d). We compared control and CBAP-KO Jurkat cells in a cell-cycle analysis using a bromodeoxyuridine (BrdU) and 7-aminoactinomycin D (7-AAD) double-labeling method and found a significant increase in the number of cells undergoing G0/G1 in CBAP-KO Jurkat cells (Fig. 1f), but no significant difference in the percentages of cells undergoing sub-G0/G1 (i.e., apoptotic) stages (Supplementary Fig. S1e) or in the number of annexin V^+^ apoptotic cells (Supplementary Fig. S1f), suggesting that CBAP KO mainly affects the cell proliferation rate in Jurkat cells. This finding is consistent with our observation that levels of bcl-2 protein, a major anti-apoptotic protein in hematopoietic cells, increased in CBAP-KO Jurkat cells (Supplementary Fig. S1g).

**Table 1:** Clinical characteristics of the eight acute lymphoblastic leukemia patients

Patient No	Age (years)	Gender	Bone marrow lymphoblast count (before treatment)	Response to Treatment	Disease-free survival (months)	Bone marrow CBAP expression^a^
P01	44	Male	65.6% (T cell)	CR to hCVAD;	9.6	++
P02	23	Male	78% (T cell)	CR to hCVAD + MTX;	1.2	++
P03	27	Male	12% (T + B cell)	CR to hCVAD	9.6	++
P04	29	Male	60% (Pre-T cell)	PD to CDVA	1.2	+
P05	9	Male	93% (T cell)	CR to TPOG-ALL	12	+
P06	24	Male	79% (T cell)	CR to hCVAD+MTX	27.6	−
P07	52	Male	66% (T cell)	CR to hCVAD	2.4	+
P08	25	Male	77% (T cell)	Unknown^b^	Unknown^b^	++

*ALL* acute lymphoblastic leukemia, *CR* complete remission, *CVAD* cyclophosphamide/vincristine/doxorubicin/dexamethasone, *hCVAD* hyper-CVAD, *CDVA* cyclophosphamide/daunomycin/vincristine/l-asparaginase, *MTX* methotrexate, *PD* progressive disease, *TPOG* Taiwan Pediatric Oncology Group

^a^All bone marrow samples were collected before any treatment regimens. The intensity of CBAP expression detected by immunohistochemical stain was defined as: “−“ for negative, “+” for weak staining, “++” for strong staining

^b^This patient was transferred to another hospital

### CBAP is involved in leukemogenesis in vivo

**Fig. 2:**
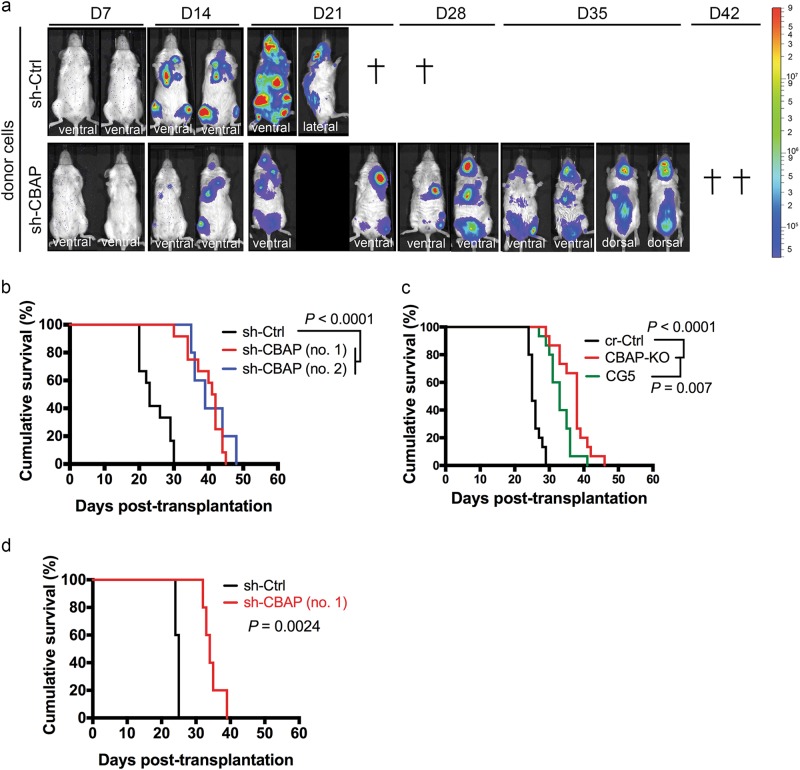
CBAP downregulation prolongs the survival of leukemic mice. **a** Tracking of leukemia outgrowth by bioluminescence imaging in NSG mice transplanted with sh-Ctrl- or sh-CBAP-expressing Jurkat cells (*n* = 2 per group). + represents mouse death. **b**–**d** Kaplan–Meier plots showing the mortality of NSG mice transplanted with various Jurkat clones (**b**, **c**) or CCRF-CEM clones (**d**) as indicated. Animal numbers in (**b**): for sh-Ctrl, *n* = 12, for sh-CBAP(no.1), *n* = 12, sh-CBAP(no.2), *n* = 5; in (**c**): cr-Ctrl, *n* = 15, CBAP-KO, *n* = 15, CG5, *n* = 15; in **d**: for sh-Ctrl, *n* = 10, sh-CBAP, *n* = 10. The survival results in **b**–**d** were compiled from experiments performed with aforementioned lines (see also results in Supplementary Fig. S3 for individual clones). The statistical significance of differences in the survival curves between groups was evaluated using the log-rank test

We next investigated the impact of CBAP loss on the leukemogenic potential of Jurkat cells in vivo. We established a leukemic mouse model by transplanting five million Jurkat cells expressing a firefly luciferase reporter gene into *NOD-SCID-IL2Rγc*-deficient (NSG) mice. At 14 and 21 days after transplantation (D), luminescence was significantly lower in mice transplanted with sh-CBAP-expressing Jurkat cells compared with control (sh-Ctrl) Jurkat cells (Fig. 2a). One mouse died at D14 and another one died at D21 in the control group (Fig. 2a, upper row). However, transplanted mice could live up to D42 under conditions of reduced CBAP expression (Fig. 2a, lower row). At D18, representative proportions of human CD45^+^ cells were significantly higher in the BM, peripheral blood leukocytes (PBLs), and spleen of mice that received control (sh-Ctrl) Jurkat cell transplants (Supplementary Fig. S2a). Leukemic mice engrafted with sh-Ctrl Jurkat cells displayed a splenomegaly phenotype (Supplementary Fig. S2b), and became moribund with severe hind limb paralysis before death. Notably, although all mice eventually succumbed to T-ALL, the mice transplanted with sh-CBAP Jurkat cells developed leukemia over a much longer latency period than those transplanted with sh-Ctrl Jurkat cells (Fig. 2b and Supplementary Fig. S3a). Mice transplanted with CBAP-KO Jurkat cells also exhibited a significantly prolonged latency period compared to those transplanted with the control (cr-Ctrl) Jurkat cells (Fig. 2c and Supplementary Fig. S3b). Introduction of CBAP-GFP fusion protein into CBAP-KO cells could partially rescue cell proliferation in tissue culture (Supplementary Fig. S3c), and leukemogenesis in vivo (Fig. 2c, green line), further supporting the importance of CBAP in leukemic cell proliferation. Moreover, shRNA knockdown of CBAP in CCRF-CEM T-ALL cells also consistently produced cells that exhibited longer disease latency in this leukemic mouse model compared to the control (Fig. 2d and Supplementary Fig. S3d), suggesting that CBAP expression increases the oncogenic activity of leukemia cells in vivo.

### Downregulation of CBAP expression in T-ALL cells results in reduced aerobic glycolysis and energy metabolism

**Fig. 3:**
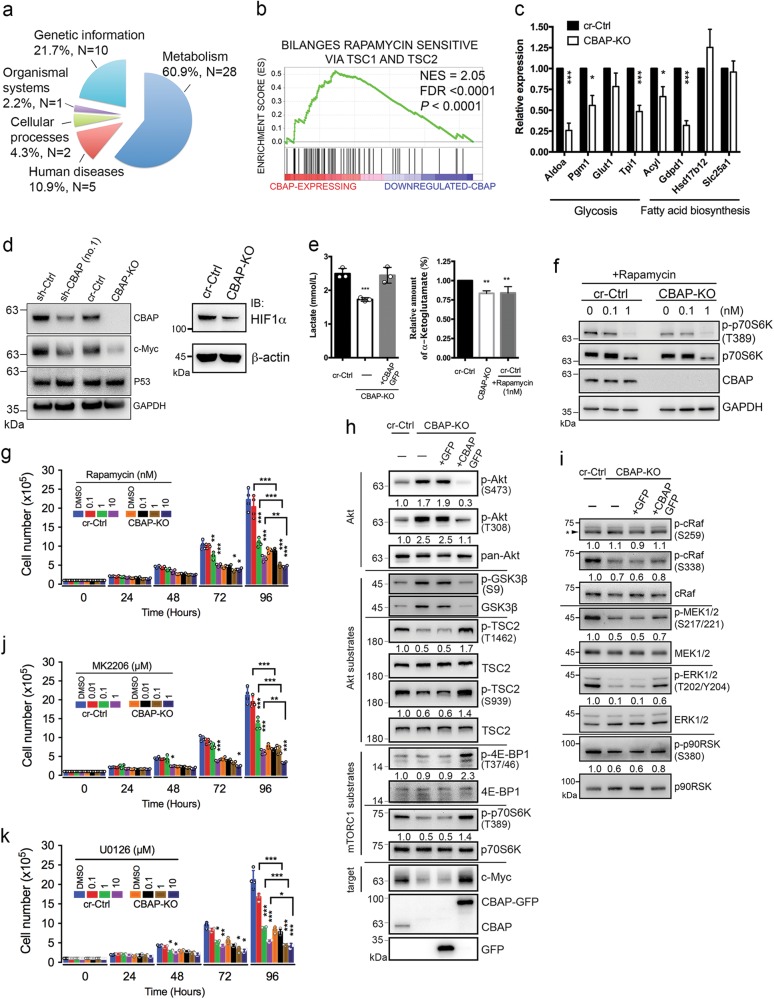
CBAP deficiency reduces aerobic glycolysis and energy metabolism and attenuates the Raf-MEK-ERK–RSK and mTORC1-S6K-4E-BP1 signaling pathways. **a** Functional categories of KEGG gene sets markedly impacted (FDR < 0.25) by CBAP downregulation. **b** Correlation of CBAP positively-regulated genes and TSC1/TSC2-dependent rapamycin-sensitive genes [26], as determined by GSEA. **c** qRT-PCR analysis of the mRNA levels of genes involved in the regulation of glycolysis and fatty acid biosynthesis. *Gapdh* was used as an internal control. The data are averages of biologically triplicated experiments. Results are plotted as mean ± SD (*n* = 3). **d** Expression of CBAP, c-Myc, P53 and HIF1α in Jurkat-derived cell clones. **e** Analysis of concentrations of lactate and α-ketoglutarate in the culture media of Jurkat and CBAP-KO cells. Data are expressed as means ± SD (n = 3; duplicate per measurement). **f** Suppression of p70S6K phosphorylation in cr-Ctrl and leukemic CBAP-KO Jurkat cells treated with the indicated doses of rapamycin for 6 h. Data represent mean ± SD of three independent experiments with duplicates of each condition. **g** Effect of the mTORC1 inhibitor, rapamycin, on Jurkat-derived leukemic cell growth. Data represent mean ± SD of three independent experiments. **h**, **i** Activation of signaling proteins involved in the Akt-TSC2-mTORC1 (**h**) and Raf-MEK-ERK (**i**) signaling pathways in the different Jurkat-derived cell lines, as indicated. Numbers under the lanes represent phosphor-proteins/total proteins normalized to that of cr-Ctrl cells (as 1.0). **j**, **k** Effects of the Akt inhibitor MK2206 (**j**) and the MEK inhibitor U0126 (**k**) on the growth of cr-Ctrl Jurkat and CBAP-KO leukemic Jurkat cells in tissue culture. Data represent mean ± SD of three independent experiments with duplicates of each condition. *P < 0.05; **P < 0.01; ***P < 0.001, according to two-tailed unpaired Student's *t* tests

We next sought to examine CBAP-regulated gene expression signatures in leukemia using a next generation sequencing technique for RNA (RNA-seq). Total mRNA was isolated from two types of CBAP-expressing Jurkat cells (sh-Ctrl cells and CBAP-KO cells expressing CBAP-GFP, with these latter referred to as CBAP-reconstituted cells) and two types of CBAP-deficient Jurkat cells (sh-CBAP cells and CBAP-KO cells expressing GFP). We then generated genome-wide transcriptome profiles for these cell lines (for method, see supplementary information). The RNA-seq data have been deposited in NCBI Gene Expression Omnibus (GEO) database (accession number: GSE 69511). Gene sets that presented highly reduced expression in the CBAP-deficient cells were related to metabolism, as assessed by KEGG (Kyoto Encyclopedia of Genes and Genomes) categories (Supplementary Table S1), and these metabolic genes represented over 60% of the significantly enriched pathways (false discovery rate (FDR) *q* value < 0.25) (Fig. 3a). Of interest, among these downregulated metabolic pathways, most of the gene sets belonged to subgroups linked to carbohydrate metabolism (35.7%) or amino acid metabolism (32.1%) (Table S1). A gene set enrichment analysis (GSEA) showed that the gene expression pattern in CBAP-deficient leukemia cells was significantly correlated with signatures of TSC1/TSC2-dependent rapamycin-sensitive genes [26] (Fig. 3b). Furthermore, the most represented genes (*P* < 0.05), i.e., pertaining to the mTORC1-induced gene set, encoded proteins involved in the regulation of glycolysis (i.e. *Aldoa, Pfkl, Pfkp, Pgm1, Glut1*, and *Tpi1*) and lipid biosynthesis (i.e. *Acly, Fasn, Gdpd1, Hsd17b12*, and *Slc25a1*) [27] (Supplementary Table S2). To further validate the results of our transcriptomic analysis, qRT-PCR experiments on expression levels of the aforementioned genes also supported that mRNA levels of many enzymes involved in glycolysis and lipid biosynthesis pathways were significantly reduced (Fig. 3c). Protein levels of two critical transcriptional factors downstream of mTORC1—c-Myc, which is important for protein translation, and HIF1α, which is important for energy metabolism—were also reduced in the CBAP-deficient Jurkat cells (Fig. 3d). Finally, critical metabolites in glycolysis (lactate) and glutaminolysis (α-ketoglutarate) were also reduced in CBAP-KO cells (Fig. 3e), suggesting that indeed the activities of several catabolic pathways such as aerobic glycolysis and glutaminolysis are diminished in the absence of CBAP proteins, resulting in reduced cell growth. These biochemical evidences prompted us to investigate whether CBAP participates in the proliferation of leukemia cells via the mTORC1 pathway since mTORC1 signaling is a well-known master regulator of protein translation, cell growth, metabolism, and cancer cell proliferation [28]. Treatment with rapamycin, a specific mTORC1 inhibitor, efficiently suppressed phosphorylation of p70S6K (a marker of mTORC1 activation, Fig. 3f) and cell growth (Fig. 3g) in both control and CBAP-KO Jurkat cell lines, demonstrating that activation of mTORC1 signaling is essential for Jurkat cell leukemic growth. Although p70S6K phosphorylation was almost completely inhibited in both control and CBAP-KO cells using 1 nM of rapamycin (Fig. 3f), loss of CBAP and rapamycin treatment had an additive effect in terms of growth suppression (Fig. 3g and Supplementary Fig. S5a). Therefore, apart from the mTORC1 signaling pathway, CBAP might act on another growth pathway (see below).

### Loss of CBAP simultaneously attenuates both Raf-MEK-ERK–RSK and mTORC1-S6K signaling

Since both PI3K-Akt and Raf–MEK–ERK signaling are reported to be crucial for mTORC1 activation [12, 14], we investigated the potential involvement of CBAP in these signaling pathways. First, CBAP KO resulted in a drastic increase of Akt phosphorylation at residues T308 and S473, whereas downstream mTORC1-S6K-4E-BP1 signaling was downregulated in CBAP-KO cells, as demonstrated by the decreased phosphorylation of TSC2 (T1462, S939) and p70S6K (T389), but not 4EBP1 (T37/46) (Fig. 3h). The relative phosphorylation of residue S9 of GSK3β compared to total GSK3β levels was not altered (Fig. 3h). Notably, compared to CBAP-KO cells, phosphorylation of TSC2, p70S6K, and 4EBP1 was all significantly enhanced in CBAP-reconstituted cells (Fig. 3h and Fig. S4 for quantification data), whereas Akt phosphorylation in these CBAP-reconstituted cells had returned to the level of control cells (Fig. 3h and Fig. S4a). These data are consistent with the hypothesis proposed by Guertin and Sabatini [28, 29] that a negative feedback inhibition loop may exist to regulate PI3K/Akt activity as a result of increased p70S6K activity. CBAP deletion also downregulated Raf-MEK-ERK signaling, as judged by decreased phosphorylation of cRaf (at residue S338, but not S259), MEK1/2 (S217/221), ERK1/2 (T202/Y204), and p90RSK (S380) (Fig. 3i, lane 2, and Supplementary Fig. S4b). Restoration of CBAP expression partially restored the phosphorylation levels of these proteins (Fig. 3i, lane 4, and Supplementary Fig. S4b). These data suggest that CBAP plays a critical role in regulating both the Akt-mTORC1 and Raf-MEK-ERK signaling pathways.

**Fig. 4:**
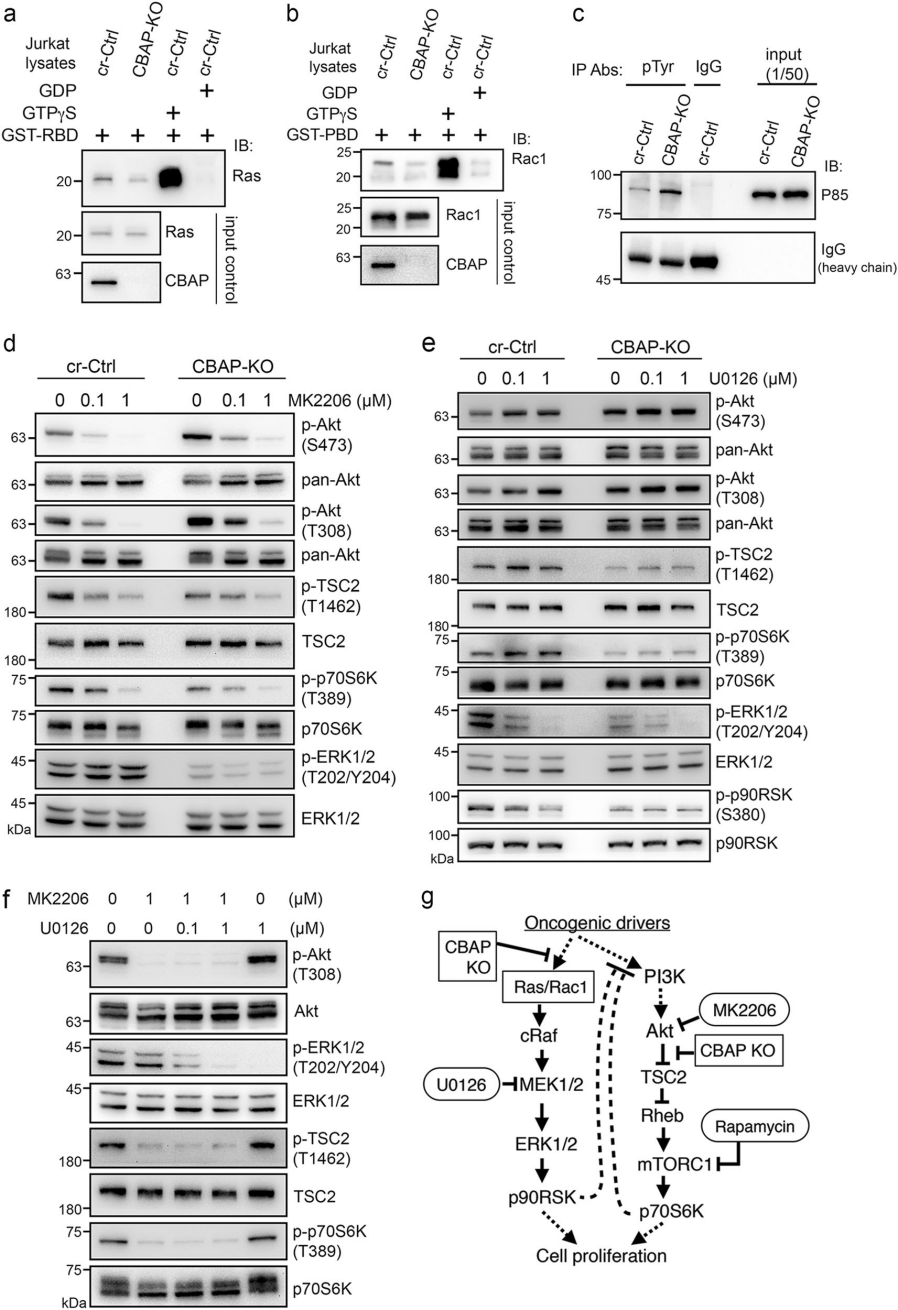
CBAP regulates upstream of Ras/Rac1 activation and TSC2-mTORC1 signaling cascades in Jurkat cells. **a**, **b** Activated Ras (**a**) and activated Rac1 (**b**) were precipitated by performing Ras-RBD and Rac1-PBD pull-down assays, respectively, and were detected by western blot using specific antibodies. Total cell lysate was pre-loaded with GTPγS or GDP as positive and negative controls, respectively, in the assays. **c** Tyrosine phosphorylation of PI3Kp85. Lysates were immunoprecipitated with anti-phosphotyrosine (pTyr) antibody before being subjected to Western blot using anti-PI3Kp85 antibody. **d**–**f** Immunoblotting analysis of signaling molecules in cr-Ctrl and leukemic CBAP-KO Jurkat cells in response to treatment with MK2206 (**d**), U0126 (**e**), or co-treatment with MK2206 and U0126 (**f**). **g** Schematic representation of the crosstalk between the Raf-MEK-ERK–RSK and Akt-TSC2-p70S6K pathways and the effects of CBAP knockout and inhibitors on the Jurkat cell line

To further investigate which signaling pathway is essential for T-ALL cell growth, we treated cells with the specific inhibitors MK2206 and U0126 that target the Akt and MEK signaling pathways, respectively. Both inhibitors effectively suppressed tumor growth in tissue cultures in a time- and dose-dependent manner (Fig. 3j, k), indicating that both pathways are important for cell growth. As for the effect of rapamycin treatment on mTORC1 activation, 1 μM MK2206 or 1 μM U0126 could almost completely inhibit Akt activity or MEK/ERK activity, respectively, in both control and CBAP-KO cells (see below, Fig. 4d, e), and loss of CBAP always had an additive effect to the inhibitor in terms of suppressing cell growth (Fig. 3j, k, see also Figs. S5b and S5c). Furthermore, suppression of leukemia cell growth in Jurkat cell-engrafted leukemic mice by either rapamycin or U0126 treatment or a combination of both further supports the importance of both the Raf-MEK-ERK and Akt-mTORC1 pathways in leukemia proliferation, especially since a combination of both inhibitors had an additive effect in vivo (Supplementary Fig. S6).

### Crosstalk between the PI3K-Akt and ERK–RSK pathways in T-ALL cells

Next, we set out to investigate the effect of CBAP on other upstream components involved in the Akt/TSC2 and Raf-MEK-ERK pathways. We measured levels of GTP-loaded Ras and Rac1 by pull-down assays with the Ras-binding domain (RBD) of Raf1 and with the p21-binding domain (PBD) of Pak1 [30], respectively. Compared to GTPγS-bound Ras (Fig. 4a) and GTPγS-bound Rac1 (Fig. 4b), only small amounts of Ras and Rac1 were GTP-loaded (lane 1 vs. lane 3). However, we found that these amounts were further reduced in CBAP-deficient cells compared to cr-Ctrl cells (lane 2 vs. lane 1). Conversely, in accordance with the effect of CBAP deficiency on Akt activity, we found that loss of CBAP increased phosphorylation of the p85 subunit of PI3K (Fig. 4c). However, it remains to be explored whether these two signaling pathways have a common upstream regulator.

ERK [31] and RSK [32] have been shown to directly act on TSC2 and mTORC1 phosphorylation, respectively, in other cancer types, and that crosstalk exists between these pathways has been demonstrated [12]. As this crosstalk may bias our interpretation of the effect of CBAP KO, we investigated the potential crosstalk between these pathways in T-ALL leukemia cells using pathway-specific inhibitors. When control cells were treated with the Akt inhibitor, MK2206, Akt phosphorylation was significantly reduced and phosphorylation levels of TSC2 and p70S6K also decreased, whereas ERK1/2 phosphorylation was not altered (Fig. 4d and Supplementary Fig. S4c for quantification data). However, when we inhibited MEK1/2 in control cells using U0126, ERK1/2 and p90RSK phosphorylation was significantly abrogated and Akt phosphorylation was increased (Fig. 4e), probably due to relief of the hypothetical cross-inhibitory signal from the Raf-MEK-ERK pathway [12]. This upregulated Akt phosphorylation in turn led to increased phosphorylation of TSC2 and p70S6K, which was especially evident when cells were treated with 0.1 μM U0126 (Fig. 4e and Supplementary Fig. S4d). Notably, increased phosphorylation of TSC2 and p70S6K was not observed in the absence of CBAP (Fig. 4e, comparing lanes 4 and 5, and Supplementary Fig. S4d) or in the co-treatment with MK2206 (Fig. 4f, comparing lane 2 to lane 5), suggesting that the crosstalk between the Raf-MEK-ERK and Akt-mTORC1 pathways occurs mainly through the Akt protein or its upstream regulator PI3-K in Jurkat cells (see Fig. 4g), but does not directly affect TSC2 or mTORC1 proteins [31, 32]. Taken together, Akt appears to be the predominant kinase controlling both TSC2 phosphorylation and downstream mTORC1 activation in leukemic Jurkat cells.

### CBAP facilitates TSC2 phosphorylation by Akt according to cell-based assays and in vitro analysis

**Fig. 5:**
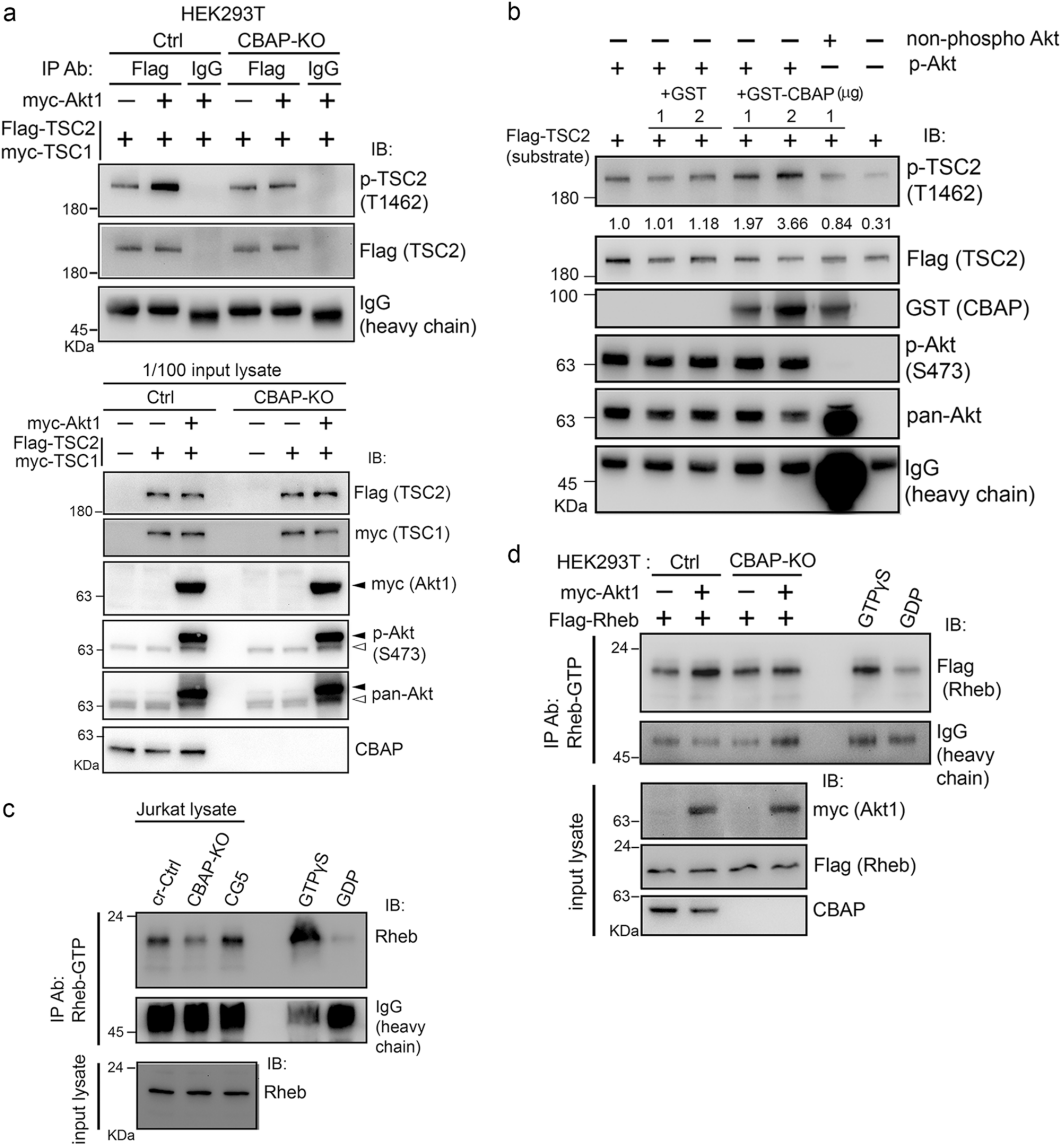
CBAP is crucial for Akt-mediated TSC2 phosphorylation and cellular Rheb-GTP levels. **a** CBAP is required for Akt-mediated TSC2 phosphorylation. Various plasmids were transfected into HEK293T cells (Ctrl or CBAP-KO). Flag-TSC2 was immunoprecipitated with anti-Flag antibody and then the immunoprecipites were subjected to immunoblotting. The input levels of proteins are shown. Black and white arrows indicate exogenous and endogenous Akt proteins, respectively. *N* = 2. **b** In vitro Akt kinase activity on TSC2 is increased in the presence of recombinant GST-CBAP protein. An in vitro Akt kinase assay was established using in vitro-translated Flag-TSC2 protein as the substrate. Phospho-Akt (S473) and non-phospho-Akt were immunopurified from CBAP-KO Jurkat cells treated with or without MK2206. Quantification of the levels of phosphorylated TSC2 normalized to total TSC2 protein is indicated above the lanes. One representative picture from two independent experiments was shown. **c** Absence of CBAP is correlated with low levels of Rheb-GTP in Jurkat cells. Endogenous Rheb-GTP levels were evaluated by immunoprecipitation using an antibody that selectively recognizes Rheb-GTP. Lysates were treated with GTPγS or GDP, which were included as positive and negative controls, respectively. **d** CBAP is essential for the Akt-induced increase in Rheb-GTP levels. Ctrl and CBAP-KO HEK293T cells were transfected with the indicated plasmids before Rheb-GTP levels were determined in the lysates as described in (**c**). Expression levels of the transfected plasmids were determined by immunoblotting using tag-specific antibodies. *N* = 2

Our aforementioned data suggest that CBAP is involved in the Akt-mTORC1 signaling pathway by controlling Akt-mediated TSC2 phosphorylation. We further investigated this dependency of Akt-mediated TSC2 phosphorylation on the presence of CBAP in HEK293T cells. We knocked out the *CBAP* gene in HEK293T cells using a CRISPR/Cas9-mediated strategy and then transiently expressed myc-Akt1 and Flag-TSC2/myc-TSC1 plasmids in this cell background (Fig. 5a, lower panel). We found the phosphorylated level of exogenously expressed Flag-TSC2 to be significantly elevated in the presence of CBAP and exogenous Akt, whereas it was not increased in HEK293T cells lacking either Akt (Fig. 5a, upper panel, lane 1) or CBAP (Fig. 5a, upper panel, lane 5), suggesting that CBAP-mediated facilitation of Akt-dependent TSC2 phosphorylation could be recapitulated in HEK293T cells using exogenous proteins. Additionally, we explored whether CBAP directly facilitates Akt-mediated TSC2 phosphorylation using an in vitro kinase assay. As shown in Fig. 5b, immunoprecipitated phospho-Akt from CBAP-KO cells possessed a limited ability to phosphorylate recombinant Flag-TSC2 protein that had been produced using an in vitro transcription/translation (IVT) system. When bacterially-derived recombinant GST-CBAP protein was added to the system, levels of phosphorylated TSC2 increased in a dose-dependent manner in vitro (Fig. 5b, lanes 4 and 5). However, when non-phosphorylated Akt was used in the in vitro kinase assay, the enhancing effect of CBAP disappeared (compare lane 4 and lane 6 in Fig. 5b), suggesting that CBAP directly activates Akt to phosphorylate TSC2. We also monitored the changing GTP-loadings of the small G protein Rheb as an independent measure of TSC2 complex inactivation by upstream Akt signals. Using an antibody that can specifically recognize GTP-loaded Rheb [33], we directly measured the nucleotide-binding states of Rheb in total cell lysates of leukemic Jurkat cells. As shown in Fig. 5c, under steady state conditions, the levels of Rheb-GTP (i.e., active Rheb) were decreased in CBAP-KO cells (lane 2) compared to both control cells (lane 1) and CBAP-reconstituted cells (CG5, lane 3). We further explored whether CBAP can regulate the levels of Rheb-GTP in the reconstituted HEK293T cells with exogenous proteins. After transfection with the Akt plasmids shown in Fig. 5d, cell lysates were immunoprecipitated with the anti-Rheb-GTP antibody and the immunoprecipitates were subjected to immunoblotting with an anti-Flag antibody. We found that there was an increase in the level of the active, GTP-bound, exogenous Flag-Rheb in control HEK293T cells (Fig. 5d, compare lanes 1 and 2). In contrast, Akt did not increase the levels of Rheb-GTP in the absence of CBAP (Fig. 5d, compare lanes 3 and 4). Thus, our data strongly suggest that CBAP can directly act on the Akt/TSC/Rheb axis to regulate downstream activation of mTORC1 signaling.

### CBAP is also required for insulin-induced TSC2 phosphorylation and translocation

**Fig. 6:**
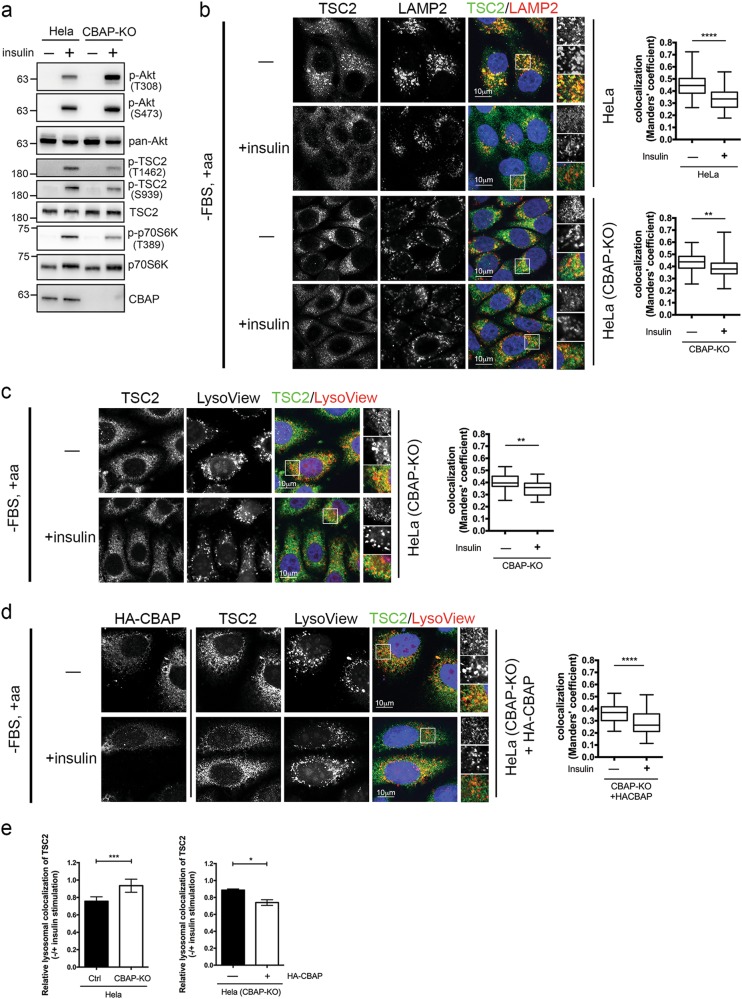
CBAP loss affects insulin-stimulated lysosomal translocation of TSC2. **a** Analysis of insulin-stimulated Akt-TSC2-p70S6K signaling activation in control and CBAP-KO Hela cells. Cells were serum starved for 16 h and then stimulated with insulin for 15 min prior to lysis. Western blots were performed using indicated antibodies. **b**–**d** Hela cells (wild type, CBAP-KO or HA-CBAP-reconstituted) were treated as in (**a**), prior to immunofluorescent co-labeling of TSC2 with LAMP2 (**b**), with LysoView (**c**), or with HA-CBAP plus LysoView (**d**). The data are representative of three independent biological replicates (*n* = 45~ 95 cells). The degree of co-localization between TSC2 and LAMP2 or LysoView is shown in the right panel. Box represents the median and interquartile range, whereas whiskers represent maximum and minimum values. For the co-localization images, representative magnified insets are shown in the rightmost panel (top: TSC2; middle: LAMP2 or LysoView; bottom: merged). **e** Relative lysosomal co-localization of TSC2 upon insulin stimulation. Statistical data were compiled from results in (**b**) (left panel), or (**c**) and (**d**) (right panel). Error bars denote ± SEM. *P < 0.05; **P < 0.01; ***P < 0.001, according to two-tailed unpaired Student's *t* tests

Since TSC2 functions as a signaling hub for many converging stimuli (including growth factors, stress, and metabolites, among others) and regulates downstream mTORC1-mediated cell metabolism, we wondered whether CBAP can also modulate Akt-dependent TSC2 phosphorylation and translocation via growth factors such as insulin. In CRISPR/Cas9-mediated CBAP-KO Hela cells, we found that insulin-stimulated phosphorylation of TSC2 and p70S6K was diminished but phosphorylation of Akt (S473 and T308) was elevated compared to control cells, suggesting that CBAP is also crucial for mediating insulin-stimulated Akt-mTORC1 signaling (Fig. 6a). Given that TSC2 phosphorylation has been reported to functionally inactivate the TSC by triggering its translocation into the cytosol and away from Rheb and mTORC1 at the lysosomal membrane [20, 34, 35], we investigated whether loss of CBAP affects TSC2 protein docking on and off the lysosomal surface in response to insulin stimulation. Through an immunostaining approach, we found that co-localization of endogenous TSC2 and LAMP2 (a well-characterized lysosomal marker) was obvious upon serum starvation and that levels of co-localization were reduced upon re-stimulation with insulin (1 μM, 15 min) in Hela (Fig. 6b, upper panel) and CBAP-KO Hela cells (Fig. 6b, lower panel). However, insulin treatment stimulated less lysosomal dissociation of TSC2 in CBAP-KO cells (Fig. 6e, left panel). Similar insulin-stimulated dissociation of TSC2 from lysosomal compartments, stained with LysoView, was also detected in CBAP-KO cells (Fig. 6c). Notably, TSC2 cycling off lysosomal compartments was profoundly restored in HA-CBAP-expressing cells in response to insulin signal (Fig. 6d, Fig. 6e, right panel). These findings show that CBAP is an integral part of the mechanism by which Akt regulates TSC2 function and localization upon growth factor stimulation.

## Discussion

This study highlights the critical role of CBAP in regulating the proliferation of T-ALL, and proposes a CBAP-dependent mode of action for Akt-mediated TSC inactivation. Our model proposes direct involvement of CBAP in Akt-dependent TSC2 phosphorylation, which facilitates dissociation of TSC2 from lysosomal surfaces, thereby relieving the Rheb-mTORC1 signaling axis from suppression by the TSC. Furthermore, loss of CBAP impairs both Rheb-mTORC1 and Raf–MEK–ERK signaling activities, resulting in reductions of leukemia cell migration and growth. Consistently, CBAP-KO T-ALL cells exhibited reduced leukemogenicity and spleen invasiveness when transplanted into immunocompromised mice. Although we cannot completely exclude the possibility of a common mechanism involving CBAP acting upstream of both the Raf-MEK-ERK and Akt-mTORC1 signaling pathways (Fig. 4g), in this study we have nevertheless clearly demonstrated that CBAP has a direct role in promoting Akt-dependent TSC2 phosphorylation, driving Rheb-mTORC1 signaling and leukemic cell growth.

Our previous studies have reported the pro-apoptotic functions of CBAP in cytokine-dependent cell lines [22] or in primary hematopoietic cells [24, 25]. However, in the current study, we have discovered an important oncogenic and pro-proliferative role of CBAP in malignant hematological cells, such as T-ALL cells. That two seemingly opposing roles for one protein occur in different cellular microenvironments is not unprecedented. The oncogene c-Myc is a well-known oncoprotein, being overexpressed in B-cell leukemia and other human cancers. However, until very recently, its apparently intrinsic apoptotic properties were not fully appreciated (Prendergast, 1999). The myeloid ecotropic viral integration site 1 (Meis1) protein coordinates with HoxA9 in vivo to drive murine myeloid leukemogenesis. However, Meis1 has an obviously pro-apoptotic function in hematopoietic cell cultures when it is expressed alone, whereas co-expression of Meis1 with HoxA9 completely suppresses cell death [36]. Similarly, Tribbles homolog 2 functions as an oncogene in inducing murine acute myelogenous leukemia (AML) and cooperates with HoxA9 to accelerate the onset of AML in vivo, which is consistent with the fact that Trib2 is highly expressed in a particular subset of human AML patients [37]. However, ectopic expression of Trib2 alone in 32D cells (an IL-3-dependent myeloid leukemia cell line) [38], or in TF-1 cells [39], induces a strong apoptotic response. Therefore, the two seemingly opposing roles of CBAP may be influenced by a “missing factor” that cooperates with CBAP to control the cellular microenvironment of host cells and determines the biological function of CBAP. This possibility warrants further investigation.

The exact mechanism of CBAP-mediated TSC2 phosphorylation remains undetermined. Previous studies have shown that CBAP is involved in ZAP70-dependent Vav1 phosphorylation and β1 integrin signalosome activation in chemokine-induced inside-out signaling [24]. It is also involved in LAT-PLCγ2-ZAP70 signalosome formation during TCR engagement [25]. We have previously mapped two discrete binding sites for Vav1 and Zap70 in the N-terminus of CBAP, as well as a β1 integrin binding site in the C-terminal half of the CBAP protein [24], but their functions remain unknown. Here we have uncovered a similar role for CBAP in facilitating TSC2 phosphorylation by Akt. Since CBAP deficiency results in a reduction of the GTP loading of multiple small G proteins such as Rac1 [24], cdc42 [24], Ras and Rheb (this report), we suspect that multiple guanine nucleotide exchange factors (GEFs) and GTPase-activating proteins (GAPs) may be regulated by CBAP. It has been reported that CBAP is a member of the Mab21 subfamily in the nucleotidyl-transferase (NTase) fold protein superfamily [23]. Proteins of this subfamily are characterized by sharing a common Mab21 core structure composed of an N-terminal NTase domain followed by a C-terminal polyA polymerase/2′−5′-oligoadenylate synthase1 (PAP/OAS1) substrate binding domain [23]. A crystallographic study of CBAP protein structure would greatly enhance our understanding of how CBAP is involved in the activation of multiple small G proteins, as well as how its structure and function diverge from those of other NTase fold protein superfamily members [23, 40].

As a therapeutic strategy targeting the mTORC1 pathway, several studies have developed single or dual inhibitors targeting components of the PI3K-Akt-mTORC1 signaling cascade and have showed profound anti-leukemic effects on T-ALL cell lines or in vivo T-ALL models, suggesting the presence of a dominant oncogenic driver in this pathway [41, 42]. The advantage of targeting CBAP as a therapeutic intervention in the PI3K-Akt-mTORC1 axis is that knockout of the *CBAP* gene in mice resulted in almost no significant acute phenotype and CBAP plays no essential role in cell metabolism or proliferation in primary cells. However, the high metabolic demands of cancer cells apparently require more efficient enzymatic complexes, which may be facilitated by overexpression of CBAP protein. Consequently, a CBAP blocker should effectively suppress cancer cell growth that is dependent on the PI3K-Akt-mTORC1 signaling axis, while leaving other primary normal cells unaffected by systemic therapy.

## Materials and methods

### Patient samples and primary cells

Human samples used in this study were approved by the Institutional Review Board (IRB; AS-IRB01-11153 and AS-IRB01-16055) of Academia Sinica (AS). Paraffin-embedded specimens of patient bone marrow were provided by Tri-Service General Hospital (Taipei, Taiwan). Informed consent has been obtained from all subjects. All cases of T-ALL and anemia non-tumor specimens were diagnosed clinically and pathologically and are detailed in Table 1. Human blood cells were separated using a Ficoll gradient and CD3^+^ T cells were sorted using a FACSAria flow cytometer (Becton Dickinson, Franklin Lakes, NJ).

### Plasmids

Plasmids expressing Flag-tagged TSC2 (#14129) and Myc-tagged TSC1 (#12133) were obtained from Addgene (Cambridge, MA). The mammalian Myc-tagged Akt1 expressing vector was generated from the pcDNA3-*Akt1* plasmid by standard PCR-assisted cloning. The full-length *Rheb* gene was cloned from the cDNA of Jurkat cells and was subclonned to generate Flag-tagged Rheb plasmid.

### Human cell lines and transplantation of NSG mice

Jurkat, MOLT-4, CCRF-CEM, HSB-2 and HL-60 cell lines were purchased from the American Type Culture Collection (ATCC, Manassas, VA). Kasumi-1 cell line was a gift from Dr. LT Hsiao obtaining from Bioresource Collection and Research Center, Taiwan. KG-1 cell line was obtained from Dr. PM Chen’s collection. For insulin stimulation, Hela cells were treated as described previously [20, 35]. NOD-scid-IL2RγKO (NSG) mice [43] were purchased from Jackson Laboratory (Bar Harbor, ME) and maintained in the specific pathogen-free animal facility of the Institute of Biomedical Sciences, AS. Animal experiments were approved by the Institutional Animal Care and Utilization Committee (IACUC) of AS (protocol number: 12-12-443). For tumor transplantation, five millions cells were transplanted by tail vein injection into 6–8-week-old male NSG recipients to generate cohorts of T-ALL leukemic mice for analyses.

### Immunohistochemistry (IHC) of patient bone marrow sections

IHC of paraffin-embedded bone marrow biopsy specimens was performed with the Cell and Tissue Staining Kit HRP-DAB System (#CTS002, CTS005, Abingdon, UK) and SignalStain Antibody Diluent (8112, Cell signaling, Danvers, MA) following the vender’s instructions.

### CRISPR/Cas9-mediated knockout and shRNA knockdown of CBAP

A CRISPR/Cas9 target site for CBAP co-expression with GFP was designed by Zgenebio Biotech Inc. (Taipei, Taiwan) (http://crispr.mit.edu). The target sequence used for CBAP was 5ʹ-GGCCCCAGCTCGGCCGCTCA-3ʹ. GFP^+^-sorted single cells were cultured to establish single-cell clones. Knockout and control (cr-Ctrl) clones were identified by immunoblot.

For shRNA-mediated knockdown of CBAP, lentiviral particles bearing specific shRNAs were provided by the National RNAi Core Facility of AS. Two different CBAP shRNAs were used: one targeting the 5′-ACCGAACGGAGAGCGAAGAAA-3′ sequence to produce the sh-CBAP (no. 1) clone, and another targeting 5′-CTGGACCTGGAATCCTGTTAC-3′ to produce the sh-CBAP (no. 2) clone. One non-targeted control shRNA (pLKO TRC025) was used to generate sh-Ctrl. Puromycin was used for selection of stable clones.

### Leukemia xenograft bioluminescent imaging

Bioluminescent imaging was performed for all animals in the cohort by intraperitoneal injection of D-Luciferin potassium salt (BioVision, Milpitas, CA) at 150 mg/kg in conscious mice. After 7 min, mice were anesthetized (1% isofluorane) for image acquisition (IVIS 200, Caliper Life Sciences, Hopkinton, MA) with a 15 s exposure. The total photon flux^−1^ for bioluminescent imaging was determined using Living Image 4.5 software (Caliper Life Sciences, Hopkinton, MA).

### Proliferation and cell-cycle analysis

Cell proliferation was evaluated using an automated cell counter (Countess, Invitrogen, Grand Island, NY) and the trypan blue exclusion method. For cell-cycle profile analysis, a BrdU/7-ADD staining kit (552598; BD Pharmingen, San Jose, CA) was used.

### Cell immuno-fluorescence, confocal microscopy and quantification of co-localization

The immuno-fluorescence experiments and calculation of Manders’ co-localization coefficient (MCC) were performed according to a previous report [35] using a Zeiss LSM 700 confocal microscope (Oberkochen, Germany). For each condition, 3–6 representative confocal images from three independent experiments were used. For calculation of Manders’ co-localization coefficient (MCC), automatic Costes thresholding was applied to analyze a total of 45–95 individual cells per condition. Statistical significance was calculated using unpaired Student’s *t* test.

### Measurement of cellular Rheb-GTP levels

To measure Rheb-GTP levels, we used a Rheb activation assay kit (81201; New East Biosciences, Malvern, PA) according to the manufacturer’s instructions.

### In vitro akt kinase assay

Akt kinase assays were carried out using a non-radioactive Akt kinase assay kit (9840; Cell Signaling Technology, Danvers, MA) according to the manufacturer’s instructions. In vitro transcription/translation (IVT; L5010, Madison, WI) Flag-TSC2 protein was synthesized and used as the substrate.

### Statistical analysis

Animal survival was assessed with Kaplan–Meier plots. The equality of the Kaplan–Meier survival distributions was assessed using the Mantel–Cox log-rank test for statistical significance. For other statistical analyses, *P* values were calculated with a two-tailed Student’s *t* test for two-group comparisons using Prism (GraphPad, La Jolla, CA). Differences were considered significant if the *P* value was < 0.05. *P < 0.05; **P < 0.01; ***P < 0.001; ****P < 0.0005.

### Cell lysis, immunoprecipitation and immunoblots

All methods for total cell lysis, immunoprecipitation, and Western blotting were described previously [24]. All antibodies used for this purpose are detailed in supplementary Table 3. The ImageJ software (Wayne Rasband, NIH) was used to quantify band intensities on the blots.

### Quantitative reverse transcription PCR

Quantitative reverse transcription PCR and cDNA synthesis were performed as described [25]. The primer sequences used for quantitative reverse transcription PCR of human CBAP were 5′-TGGCAAAGCTCTGTAGACCA-3′ (forward) and 5′-CTGGGCGGAACATAATGTGG-3′ (reverse). Relative fold expression was calculated by the 2^−ΔΔ*C*^_T_ method [44].

## Electronic supplementary material

Supplementary information
