# Supplementary material for: CBAP modulates Akt-dependent TSC2 phosphorylation to promote Rheb-mTORC1 signaling and growth of T-cell acute lymphoblastic leukemia
Source: Oncogene. 2018 Sep 28;38(9):1432–47. doi: 10.1038/s41388-018-0507-6 (PMC6372575; doi:10.1038/s41388-018-0507-6)
Supplement: Supplementary file 1 — Supplementary information [file 41388_2018_507_MOESM1_ESM.pdf]

CBAP modulates Akt-dependent TSC2 phosphorylation to promote Rheb-mTORC1 signaling and growth of T-cell acute lymphoblastic leukemia

SUPPLEMENTAL INFORMATION

In vivo drug preparation and Jurkat leukemia xenograft bioluminescent imaging.

1 mg of Rapamycin or U0126 was dissolved in 20 μl of ethanol and then was diluted with PBS to a final concentration of 0.75 mg/ml directly before use. One day after transplantation of Jurkat T-ALL cells (5× 10^6 cells; stable expression of pGL4.1-luciferase reporter gene), leukemic mice were administrated daily intraperitoneal injection of vehicle or drugs (rapamycin (7.5 mg/kg), U0126 (7.5 mg/kg) or combined dosage) for 7 days. Bioluminescent imaging was performed on days 9 and 14 by intraperitoneal injection of D-Luciferin potassium salt (BioVision) at 150 mg/kg in awake mice. After 7 min of distribution, mice were anesthetized (1% isofluorane) for imaging acquisition (IVIS 200, Caliper Life Sciences) with a 15 sec exposure. The total photon $flux^{-1}$ of bioluminescent imaging was determined using Living Image 4.5 software (Caliper Life Sciences).

Flow cytometry assay

Single-cell suspensions, cell staining and flow cytometry were as described previously [1] and data were evaluated with FlowJo software (TreeStar, Ashland, OR).

In vitro chemotaxis assays and treatment

In vitro chemotaxis assays were performed as described [1].

RNA preparation, next generation sequencing and bioinformatics

Total RNA was extracted from cells using the RNeasy Mini kit (Qiagen, Venlo, Netherland). RNA quality was determined with a Bioanalyzer (Agilent, Santa Clare, CA), which showed RNA integrity >9.5 for all samples. Samples were sequenced with an Illumina HiSeq 2000

platform using 100-bp paired-end reads. Fasq files were aligned to version 19 of the human genome (hg19) using TopHat 2. The cuffdiff module of Cufflinks (http://cole-trapnell-lab.github.io/cufflinks/) was used to estimate the fragments per kilobase of exon model per million mapped fragments (FPKMs) and the differentially expressed transcripts. GSEA was performed using gene set as the permutation type, with 1000 permutations and the $\log_2$ ratio of classes as the metric for ranking genes. Gene sets used in this study were identified from the Molecular Signatures Database (MSigDB Curated v3.0). The RNA-seq data have been deposited in NCBI Gene Expression Omnibus (GEO) database (accession number: GSE 69511).

References:

1. Chiang YJ, Ho KC, Sun CT, Chiu JJ, Lee FJ, Liao F, et al. CBAP functions as a novel component in chemokine-induced ZAP70-mediated T-cell adhesion and migration. PLoS One. 2013;8:e61761.

SUPPLEMENTARY FIGURES

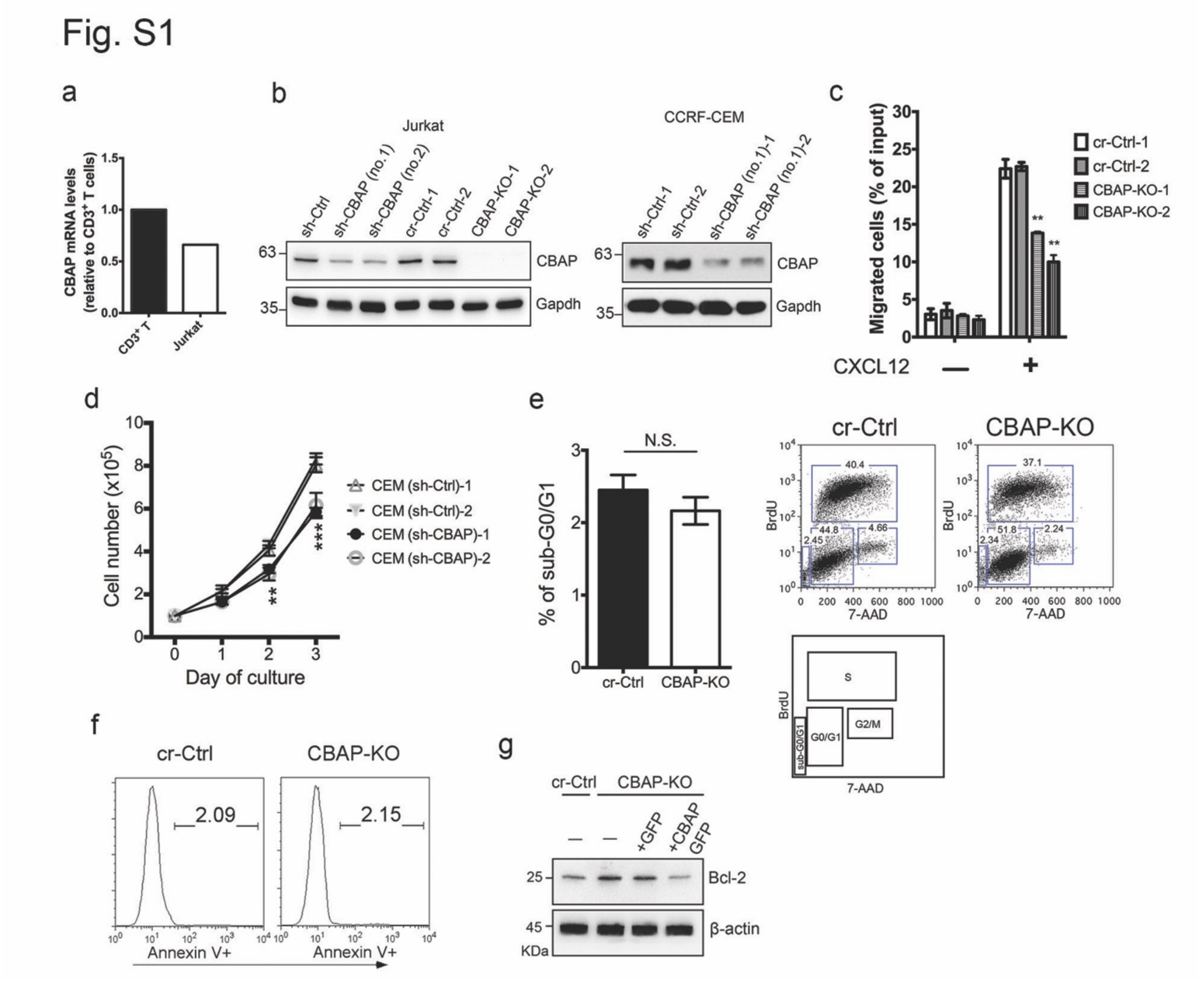

Supplementary Fig. S1 Expression of *CBAP mRNA* and protein and its effect on migratory activity, cell growth, and apoptosis in human T-ALL cell lines. **a** Comparison of *CBAP* mRNA levels in human peripheral T lymphocytes ($CD3^+$) and Jurkat T-ALL cells by quantitative PCR (normalized to GAPDH). **b** Expression of CBAP in independent CBAP shRNA knockdowns (sh-CBAP no. 1 or no. 2) and CRISPR/Cas9-mediated CBAP knockout (CBAP-KO-1 and CBAP-KO-2) lines and their respective controls (sh-Ctrl, cr-Ctrl) in Jurkat cells or CCRF-CEM cells. **c** CXCL12-induced transwell migration of CBAP-KO Jurkat clones compared to cr-Ctrl clones. **d** Growth curves of CCRF-CEM cells knocked down with CBAP shRNA (sh-CBAP no. 1) or sh-Ctrl. Two independent clones per group are shown. **e**

Representative dot plots and gating profiles for the frequency of sub-G0/G1 cells. Control (cr-Ctrl) and CBAP-KO Jurkat cells were stained using BrdU/7-ADD. **f** Representative data showing the frequency of apoptosis in cr-Ctrl- and CBAP-KO Jurkat cells determined by annexin V staining. **g** Western blot of Bcl2 protein in indicated Jurkat clones.

Fig. S2

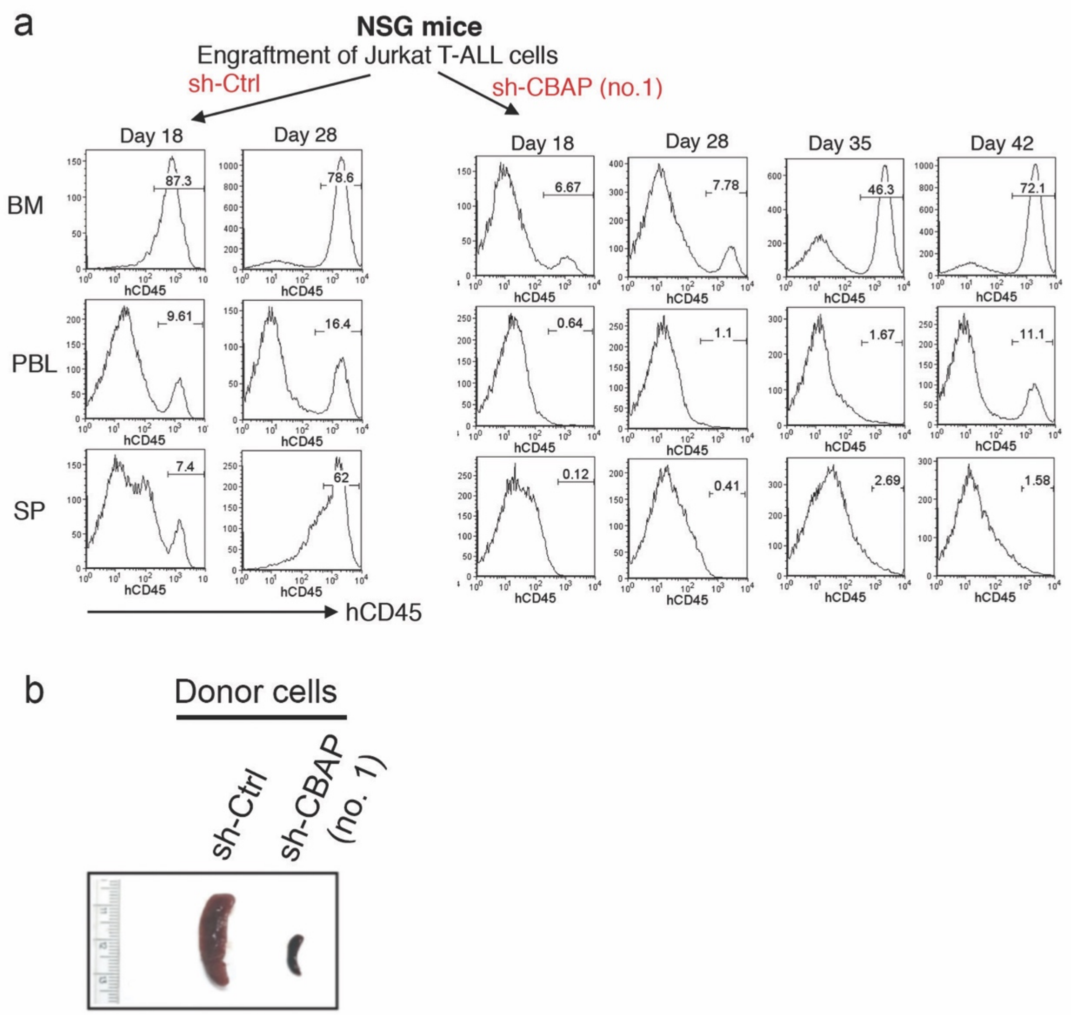

Supplementary Fig. S2 CBAP knockdown reduces leukemogenesis *in vivo.* **a** Tracking of human $CD45^+$ leukemia cells by immunostaining and FACS analysis of cells obtained from bone marrow (BM), peripheral blood leukocytes (PBL), and the spleen (SP) of recipient mice on the indicated days after transplantation with sh-Ctrl- or sh-CBAP (no. 1)-expressing Jurkat cells. The percentage of $CD45^+$ leukemia cells is shown in each panel. **b** Spleen size in recipient mice 28 days after transplantation with the indicated Jurkat T-ALL cell lines.

Fig. S3

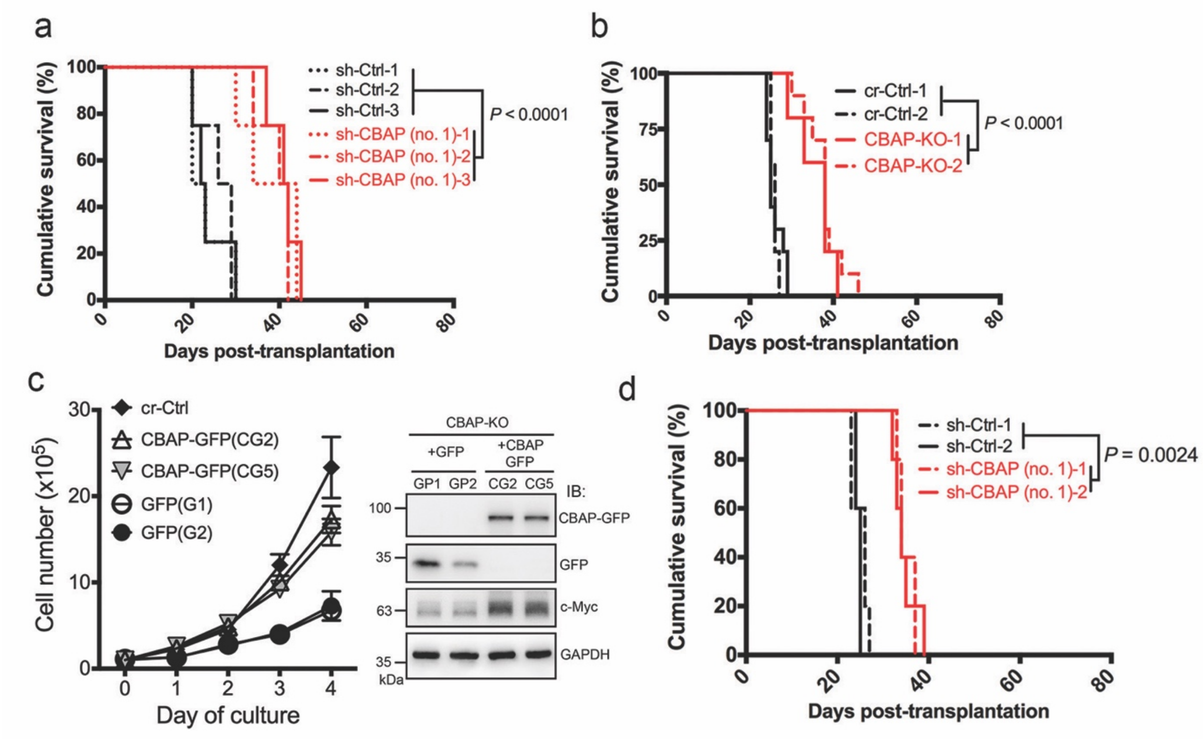

Supplementary Fig. S3 CBAP downregulation extends the survival of leukemic mice. Kaplan-Meier plots representing survival of NSG mice after transplantation with various T-ALL independent clones. **a** Jurkat clones stably transduced with lentiviruses expressing a control sh-RNA (sh-Ctrl, 3 independent clones, n = 4 per black line) or CBAP sh-RNA (sh-CBAP no.1, 3 independent clones, n = 4 per red line). **b** Jurkat clones modified by CRISPR/Cas9 plasmids without (cr-Ctrl-1, n-5; cr-Ctrl-2, n-10), or with, knockout of CBAP (CBAP-KO-1, n=5; CBAP-KO-2, n=10). **c** Partial rescue of cell growth by a CBAP-GFP fusion gene. Jurkat leukemia stable clones, including cr-Ctrl and CBAP-KO lines with stable expression of GFP (GP1 and GP2) or the CBAP-GFP fusion gene (CG2 and CG5) were cultured. Specific protein expression was determined by immunoblotting using indicated antibodies. **d** Survival of NSG recipients engrafted with CCRF-CEM leukemia cells silenced with control sh-RNA (sh-Ctrl, 2 independent clones, n = 5 per black line) or CBAP sh-RNA (sh-CBAP no. 1, 2 independent clones, n = 5 per red line).

Fig. S4

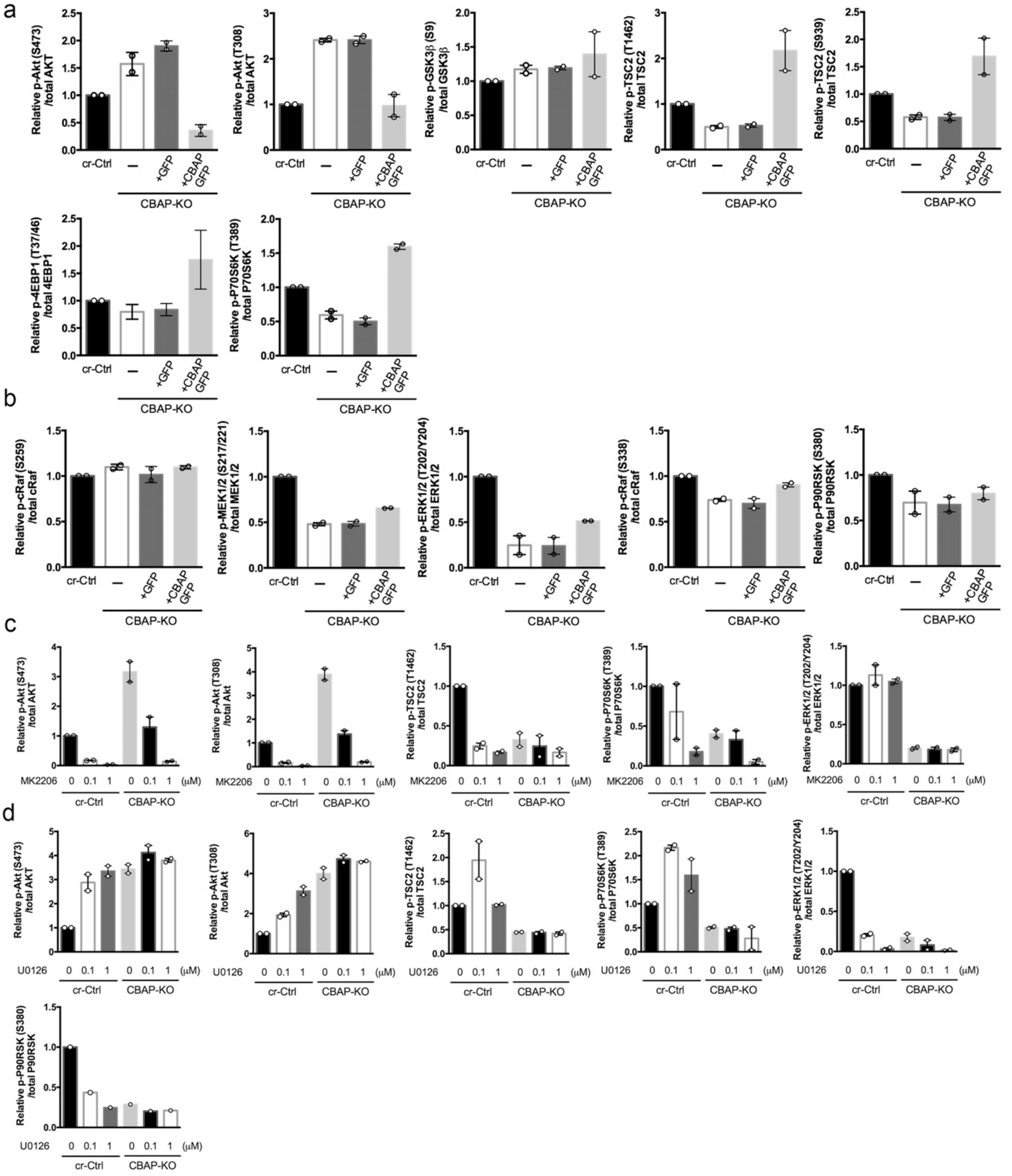

Supplementary Fig. S4 a, b, c, d Bar graphs illustrating relative phosphorylation of indicated proteins from Fig. 3h **a**, Fig. 3i **b**, Fig. 4d **c**, and Fig. 4e **d**. Phospho-protein level of cr-Ctrl cells was used as 1 for per group. Two independent experiments were used, except for p-P90RSK (n=1) in **d**. Error bars represent the SD of mean.

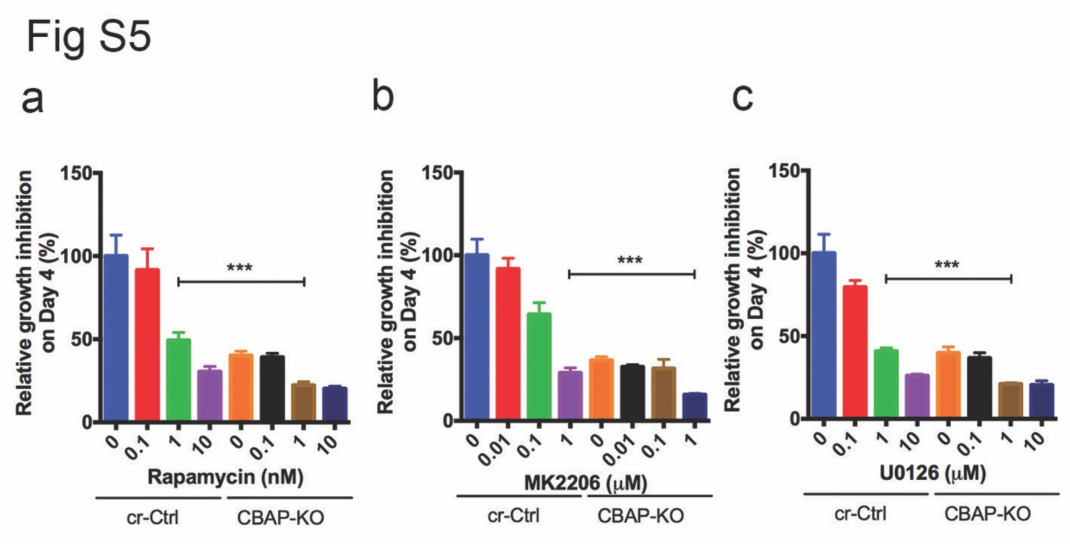

Supplementary Fig. S5 Relative fold change of cell growth on Day 4 after treatment with inhibitors from results of Fig 3. g, j and k.

Fig. S6

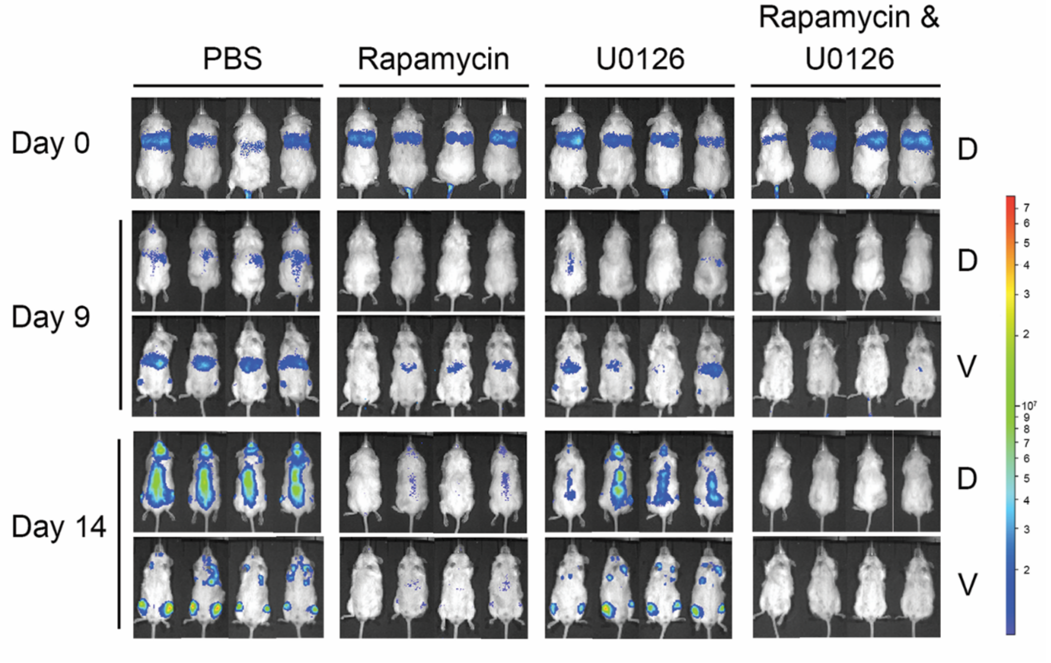

Supplementary Fig. S6 Sequential bioluminescent imaging of Jurkat T-ALL-transplanted mice with *in vivo* administration of vehicle (PBS), rapamycin (7.5 mg per kg body weight), U0126 (7.5 mg per body weight) or combined drugs. The scale on the right shows the color scheme for low (blue) to high (red) photon flux. D, stands for dorsal; V, stands for ventral.

Supplementary Table S1 Genes sets in KEGG categories affected by down-regulation of CBAP expression

KEGG category	KEGG pathway	Number of genes in gene list	Number of those genes in our gene list	NES	p-value	FDR
Metabolism	Carbohydrate metabolism					
	KEGG_GLYCOLYSIS_GLUCONEOGENESIS	62	36	1.8638	0.0016	7.91E-03
	KEGG_CITRATE_CYCLE_TCA_CYCLE	32	28	1.8998	<0.001	5.18E-03
	KEGG_PENTOSE_AND_GLUCURONATE_INTERCONVERSIONS	28	10	1.6000	0.0265	5.27E-02
	KEGG_PROPANOATE_METABOLISM	33	26	1.8492	0.0032	7.33E-03
	KEGG_BUTANOATE_METABOLISM	34	23	2.0784	<0.001	5.21E-04
	KEGG_PYRUVATE_METABOLISM	40	28	1.7785	0.0016	1.35E-02
	KEGG_FRUCTOSE_AND_MANNOSE_METABOLISM	34	27	1.7027	0.0033	2.55E-02
	KEGG_AMINO_SUGAR_AND_NUCLEOTIDE_SUGAR_METABOLISM	44	38	1.6627	0.0063	3.15E-02
	KEGG_GALACTOSE_METABOLISM	26	19	1.6407	0.0195	3.74E-02
	KEGG_GLYOXYLATE_AND_DICARBOXYLATE_METABOLISM	16	13	1.5138	0.0386	8.53E-02
	Nucleotide metabolism					
	KEGG_PYRIMIDINE_METABOLISM	98	81	1.8135	0.0015	1.02E-02
	KEGG_PURINE_METABOLISM	159	113	1.4892	0.0098	1.00E-01
	Amino acid metabolism					
	KEGG_VALINE_LEUCINE_AND_ISOLEUCINE_DEGRADATION	44	38	2.0277	<0.001	6.68E-04
	KEGG_ARGININE_AND_PROLINE_METABOLISM	54	37	1.8173	0.0015	1.03E-02
	KEGG_GLYCINE_SERINE_AND_THREONINE_METABOLISM	31	19	1.7385	0.0066	1.75E-02
	KEGG_VALINE_LEUCINE_AND_ISOLEUCINE_BIOSYNTHESIS	11	9	1.6889	0.0119	2.67E-02
	KEGG_TYROSINE_METABOLISM	42	20	1.5689	0.0265	6.04E-02
	KEGG_PHENYLALANINE_METABOLISM	18	8	1.4450	0.0793	1.34E-01
	KEGG_LYSINE_DEGRADATION	44	39	1.3766	0.0806	1.70E-01
	KEGG_TRYPTOPHAN_METABOLISM	40	20	1.3672	0.0927	1.77E-01
	KEGG_ALANINE_ASPARTATE_AND_GLUTAMATE_METABOLISM	32	23	1.3481	0.1190	1.88E-01
	Global and overview maps					
	KEGG_FATTY_ACID_METABOLISM	42	28	1.4193	0.0649	1.47E-01
	Energy metabolism					
	KEGG_OXIDATIVE_PHOSPHORYLATION	135	100	2.0733	<0.001	8.00E-04
	Metabolism of cofactors and vitamins					
	KEGG_PORPHYRIN_AND_CHLOROPHYLL_METABOLISM	41	19	1.6764	0.0176	2.89E-02
	KEGG_ONE_CARBON_POOL_BY_FOLATE	17	13	1.3909	0.1020	1.59E-01
	Metabolism of other amine acids					
	KEGG_BETA_ALANINE_METABOLISM	22	16	1.5446	0.0428	7.04E-02
	Xenobiotics biodegradation and metabolism					
	KEGG_DRUG_METABOLISM_OTHER_ENZYMES	51	18	1.4087	0.0867	1.48E-01
	Metabolism of terpenoids and polyketides					
	KEGG_LIMONENE_AND_PINENE_DEGRADATION	10	7	1.3615	0.1396	1.78E-01
Human Diseases	Neurodegenerative diseases					
	KEGG_PARKINSONS_DISEASE	133	97	2.0426	<0.001	8.02E-04
	KEGG_HUNTINGTONS_DISEASE	185	143	1.7501	<0.001	1.60E-02
	KEGG_ALZHEIMERS_DISEASE	169	128	1.5694	0.0041	6.24E-02
	Immune diseases					
	KEGG_PRIMARY_IMMUNODEFICIENCY	35	26	1.5140	0.0465	8.80E-02
	KEGG_SYSTEMIC_LUPUS_ERYTHEMATOSUS	140	60	1.5835	0.0127	5.73E-02
Cellular processes	Transport and catabolism					
	KEGG_PEROXISOME	78	60	1.4395	0.0358	1.35E-01
	Cellular community					
	KEGG_TIGHT_JUNCTION	134	83	1.3254	0.0598	2.12E-01
Organismal systems	Immune system					
	KEGG_ANTIGEN_PROCESSING_AND_PRESENTATION	89	43	1.4005	0.0612	1.53E-01
Genetic information processing	Transcription					
	KEGG_RNA_POLYMERASE	29	28	2.1614	<0.001	0.00E+00
	KEGG_SPLICEOSOME	128	119	1.4103	0.0243	1.51E-01
	Translation					
	KEGG_RIBOSOME	88	82	1.9352	<0.001	3.64E-03
	KEGG_AMINOACYL_TRNA_BIOSYNTHESIS	41	41	1.8611	0.0016	7.12E-03
	Folding, sorting and degradation					
	KEGG_PROTEASOME	48	42	2.2196	<0.001	0.00E+00
	Replication and repair					
	KEGG_DNA_REPLICATION	36	34	1.8013	0.0015	1.13E-02
	KEGG_NUCLEOTIDE_EXCISION_REPAIR	44	41	1.7695	0.0016	1.39E-02
	KEGG_MISMATCH_REPAIR	23	21	1.6906	0.0051	2.77E-02
	KEGG_BASE_EXCISION_REPAIR					
	KEGG_NON_HOMOLOGOUS_END_JOINING	14	13	1.3135	0.1594	2.22E-01

Supplementary Table S2 Log_2-transformed fold changes in FPKM expression values between CBAP-expressing and downregulated CBAP group genes involved in mTORC1-regulated metabolic pathways.

Gene symbol	Fold change (log_2)
**Glycolysis**	
Aldoa	-0.233
Bpgm	-0.063
Hk1	-0.093
Hk2	-0.361
Pdk1	-0.090
Pfkl	-0.207
Pfkp	-0.424
Pgk1	0.041
Pgm1	-0.133
Pgm2	-0.006
Slc2a1/Glut1	-0.476
Tpi1	-0.585
**Pentose pathway**	
G6pd	0.189
Pgd	0.188
Rpe	0.067
Rpia	0.158
Taldo1	-0.169
**Lipid biosynthesis**	
Acly	-0.349
Acsl3	0.649
Acss2/Acas2	-0.002
Agpat5	-0.088
Elovl1	-0.119
Elovl5	-0.165
Fasn	-0.797
Gdpd1	0.587
Ggps1	0.143
Hsd17b12	0.177
Hsd17b7	-0.289
Mvk	-0.428
Slc25a1	-0.627
Soat1	-0.001

Supplementary Table S3 List of antibodies and reagents

ANTIBODIES & REAGENTS	SOURCE	IDENTIFIER
Flag M2 affinity gel	Sigma-Aldrich (St. Louis, MO)	A2220
Flag tag	Sigma-Aldrich (St. Louis, MO)	F7425
Akt1/2/3	Abcam (Cambridge, MA)	ab179463
p-Akt1 (S473)	Abcam (Cambridge, MA)	ab81283
GSK3β	Abcam (Cambridge, MA)	ab32391
p-GSK3β (S9)	Abcam (Cambridge, MA)	ab75814
TSC2	Abcam (Cambridge, MA)	ab52936
Rheb	Abcam (Cambridge, MA)	ab92313
p70S6K	Abcam (Cambridge, MA)	ab9202
p-p70S6K (T389)	Abcam (Cambridge, MA)	ab19380
LAMP2	Abcam (Cambridge, MA)	ab25631
rabbit-DyLight-488	Abcam (Cambridge, MA)	ab96899
mouse-DyLight-594	Abcam (Cambridge, MA)	ab96881
actin	Cell Signaling Technology (Danvers, MA)	3700
c-Myc	Cell Signaling Technology (Danvers, MA)	5605
Myc-tag	Cell Signaling Technology (Danvers, MA)	2276
TSC2	Cell Signaling Technology (Danvers, MA)	4308
p-TSC2 (S939	Cell Signaling Technology (Danvers, MA)	3615
p-TSC2 (T1462)	Cell Signaling Technology (Danvers, MA)	3617
p-4E-BP1 (T37/46)	Cell Signaling Technology (Danvers, MA)	2855
p-Akt (T308)	Cell Signaling Technology (Danvers, MA)	2965
p-cRaf (S259)	Cell Signaling Technology (Danvers, MA)	9421
p-cRaf (S338)	Cell Signaling Technology (Danvers, MA)	9427
p-MEK1/2 (S217/221)	Cell Signaling Technology (Danvers, MA)	9121
MEK1/2	Cell Signaling Technology (Danvers, MA)	8727
p-ERK1/2 (T202/Y204)	Cell Signaling Technology (Danvers, MA)	4370
ERK1/2	Cell Signaling Technology (Danvers, MA)	4695
p-p90RSK (S380)	Cell Signaling Technology (Danvers, MA)	11989
p90RSK	Cell Signaling Technology (Danvers, MA)	9355
TSC1	Cell Signaling Technology (Danvers, MA)	6935
TBC1D7	Cell Signaling Technology (Danvers, MA)	14949
CD3ε	Cell Signaling Technology (Danvers, MA)	85061
TSC2	Cell Signaling Technology (Danvers, MA)	4308
4E-BP1	GeneTex (Irvine, CA)	GTX50543
GFP	GeneTex (Irvine, CA)	GTX113617
HA tag	GeneTex (Irvine, CA)	GTX115044;GTX628489
GFP	Clontech (Mountain View, CA)	632381
Active-Rheb (Rheb-GTP)	NewEast Biosciences (Malvern, PA)	26910
LysoView-633 dye	Biotium	70058

Supplementary Table S4 Primer sequences for quantitative PCR of genes involved in glycolysis and fatty acid biosynthesis

**Glycolysis**	Forward primer	Reverse primer	Prod. Size (bp)
Aldolase, Fructose-Bisphosphate A (*Aldoa*)	GACACTCTACCAGAAGGCGGAT	GGTGGTAGTCTCGCCATTTGTC	130
Phosphofructokinase, Liver Type (*Pfkl*)	AAGAAGTAGGCTGGCACGACGT	GCGGATGTTCTCCACAATGGAC	113
Phosphofructokinase, platelet (*Pfkp*)	AGGCAGTCATCGCCTTGCTAGA	ATCGCCTTCTGCACATCCTGAG	127
Phosphoglucomutase 1 (*Pgm1*)	TTGCAGGCTTCTCCAACTGGAC	ATGTCCTCCACACTCTGCTTGC	142
Glucose transporter 1 (*Glut1*)	TTGCAGGCTTCTCCAACTGGAC	CAGAACCAGGAGCACAGTGAAG	113
Triosephosphate isomerase (*Tpi1*)	CGAGCAGACAAAGGTCATCGCA	TCGGAGCTTCTCGTGTACTTCC	136
**Fatty acid biosynthesis**			
ATP citrate lyase (*Acly*)	GCTCTGCCTATGACAGCACCAT	GTCCGATGATGGTCACTCCCTT	141
Fatty acid synthase (*Fasn*)	TTCTACGGCTCCACGCTCTTCC	GAAGAGTCTTCGTCAGCCAGGA	131
Glycerophosphodiester phosphodiesterase domain containing 1 (*GDPD1*)	GCACCAGAGAAAGAAGCAGCGA	CGCATGCTGAAAGGCTGCCATT	100
Hydroxysteroid 17-Beta Dehydrogenase 12 (*HSD17B12*)	GCCAACTTTGGATAAGCCCTCTC	AGGCAGGTTTGAGATTATCGAGC	127
Solute carrier family 25 member 1 (*SLC25A1*)	GTGAAGTTCATCCACGACCAGAC	TGCTTCAGGACAGTGGCTGTGA	131
